# Sudden death in young persons with uncontrolled asthma - a nationwide cohort study in Denmark

**DOI:** 10.1186/s12890-015-0033-z

**Published:** 2015-04-14

**Authors:** Anders Juul Gullach, Bjarke Risgaard, Thomas Hadberg Lynge, Reza Jabbari, Charlotte Glinge, Stig Haunsø, Vibeke Backer, Bo Gregers Winkel, Jacob Tfelt-Hansen

**Affiliations:** Danish National Research Foundation Centre for Cardiac Arrhythmia (DARC), University of Copenhagen, Copenhagen, Denmark; Laboratory of Molecular Cardiology, Department of Cardiology, The Heart Centre, Copenhagen University Hospital, Rigshospitalet, 9312, Juliane Maries Vej 20, Copenhagen, 2100 Denmark; Department of Medicine and Surgery, University of Copenhagen, Copenhagen, Denmark; Department of Respiratory Medicine, Bispebjerg University Hospital, Copenhagen, Denmark

**Keywords:** Sudden unexpected death, Asthma, Causes of sudden unexpected death, Death, Signs and symptoms, Clinical characteristics

## Abstract

**Background:**

Asthma is a common chronic disease among young adults, and several studies have reported increased mortality rates in patients with asthma. However, no study has described sudden unexpected death in a nationwide setting in patients with uncontrolled asthma. We defined uncontrolled asthma as a previous hospital admittance because of asthma (of any severity) or when asthma was considered to have influenced the death according to the death certificate. The purpose of this study is to increase the medical focus on young persons with uncontrolled asthma and thereby hopefully aid in preventing sudden unexpected deaths. We therefore aimed to describe clinical characteristics, symptoms, causes of death, and contact with the healthcare system prior to sudden unexpected death in young persons with uncontrolled asthma.

**Methods:**

Through the review of death certificates, we found 625 sudden unexpected death cases in individuals aged 1–35 years in Denmark from 2000 to 2006. Of those, 49 persons with uncontrolled asthma were identified.

Previous contacts with the healthcare system were identified, and available records from general practitioners were retrieved.

**Results:**

We identified 49 individuals who suffered from uncontrolled asthma. This corresponds to an incidence rate of 0.32 per 100,000 person-years. The cause of death in 31 cases (63%) was sudden cardiac death, and in 13 cases (27%), it was a fatal asthma attack.

Symptoms (chest pain, dyspnea, seizures, general malaise, syncope, and palpitations) prior to death were reported in 41 (84%) of the cases. In 34 (69%) of the cases, antecedent symptoms (symptoms >24 hours before death) were present, and 28 (57%) patients had prodromal symptoms (symptoms <24 hours before death). The most common antecedent symptoms were dyspnea and chest pain, whereas the most common prodromal symptoms were dyspnea, general malaise, and/or fatigue. Twenty-eight patients (57%) sought medical advice from a general practitioner and/or emergency department due to these symptoms.

**Conclusion:**

The cause of death was predominantly sudden cardiac death followed by fatal asthma attack. We found that 41 (84%) of patients suffered from symptoms prior to death and that 28 (57%) sought medical advice from the emergency department and/or general practitioners.

**Electronic supplementary material:**

The online version of this article (doi:10.1186/s12890-015-0033-z) contains supplementary material, which is available to authorized users.

## Background

Asthma is a variable chronic inflammatory disease of the airways that causes changeable airway hyperresponsiveness leading to inconstant airflow obstruction [1]. The prevalence of asthma has increased over the past decades [1-5], and it is one of the most common chronic diseases among young adults in Western society. Asthma can lead to reduced quality of life for the individual and increased healthcare costs for the society, and it has been shown that pupils with asthma miss 2.1-14.8 more days of school compared with non-asthmatic pupils [6].

Several studies have reported increased mortality rates in patients with asthma. In a 7-year follow-up cohort of 3,880 individuals randomly selected from a background population, the presence of asthma attacks and nocturnal symptoms was associated with a two-fold increased risk of death by all causes [4]. Similarly, a Danish 17-year follow-up study of 13,540 individuals 20 years of age or older with self-reported asthma who were randomly selected from the general population revealed a gender-independent slight increase in mortality rate compared with individuals without asthma [7].

The incidence rate of sudden unexpected death (SD) in children and young adults (1–35 years) is 3.75 per 100,000 person-years, which corresponds to approximately 10% of all deaths in this age group [8]. Asthma is a well-known risk factor for SD in the presence of other diseases [3,9]; for example, in patients with long QT syndrome, asthma is associated with an increased risk of cardiac events even when adjusted for β-mimetic therapy [9]. Furthermore, it has been described that young SD asthma patients exhibited a general fragmentation of the AV bundle upon autopsy, suggesting that an arrhythmic event led to SD [3].

To our knowledge, no study has previously described the causes of SD in a nationwide setting in patients with uncontrolled asthma. We aimed to identify and characterize the symptoms before SD in young persons with uncontrolled asthma and to describe the causes of SD.

## Methods

### Study design and study population

**Figure 1:**
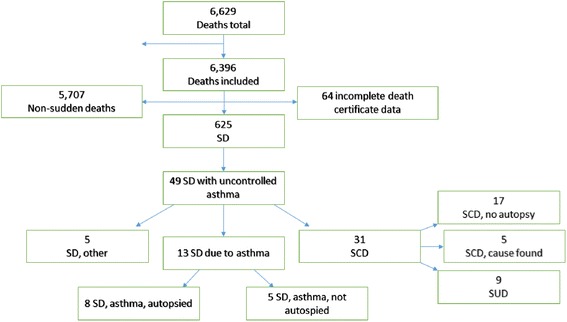
Cause of death, flowchart.

We have previously identified all SD cases in individuals aged 1–35 years in Denmark from 2000 to 2006 [8]. In brief, this was a nationwide retrospective study using the availability of all death certificates and the registration of all inpatient and outpatient activity in Danish hospitals and emergency departments (ED) together with access to all medical records and autopsy reports, as previously described in detail by Winkel et al. [8]. All death certificates were retrieved electronically and were read independently by two physicians to identify all SD cases. For the purpose of this study, we included only those SD cases occurring in persons with uncontrolled asthma (see Figure 1).

The study was approved by The Ethics Committee of the Capital Region of Denmark (H-KF-27284), the Danish Data Protection Agency (2011-41-5767), and the Danish National Board of Health (7-505-29-58/6).

### Danish registries and death certificates

All Danish citizens have a unique civil registration number, which can be linked to all healthcare-related contacts. Using the civil registration number, we retrieved information on prior medical history from the National Patient Registry. All diagnoses are coded according to the International Classification of Diseases (ICD) [10], with ICD-8 codes used from 1977 and ICD-10 codes used since 1994 [11].

Danish death certificates can only be issued by a medical doctor and are always issued if the death occurs within Danish borders (at home or at the hospital). The death certificate contains a highly informative supplementary information field that describes in detail the circumstances relating to the death [8,12,13]. This information field is mandatory in all medico-legal external examinations. In cases where a person is found dead and the death is sudden and unexpected, external examinations are mandatory by law. The external examination is performed by the police and one of the 34 medical officers of public health (certified medical doctor). The medical officer of public health always has access to 1) first responder (emergency medical service) records, 2) the medical files related to the death (if any), 3) the entire police record including all witness statements, and 4) the body.

All our autopsied sudden cardiac death (SCD) cases were identified based on an evaluation by two forensic pathologists to determine the presence of conduction abnormalities, myocarditis, hypertrophic cardiomyopathy, etc. [8].

### Data collection

Death certificates and autopsy reports were read for all SD cases occurring in individuals with uncontrolled asthma. Using the civil registration number, the general practitioner (GP) of the deceased was identified, and the full medical record from the GP and hospital was retrieved manually as previously described [14-16].

### Definitions

SD was defined as a sudden, natural, unexpected death; in *witnessed* cases, this was defined as an acute change in cardiovascular status with time to death being < 1 hour, and in *unwitnessed* cases, SD was defined as a person last seen alive and functioning normally < 24 hours before being found dead [8].

For the purpose of this study, persons were considered to have uncontrolled asthma when previously admitted to the hospital because of any severity of asthma (ICD-10 code J45 and J46) or when asthma was thought to have influenced the death according to the death certificate.

Symptoms prior to death were defined as chest pain (chest tightness or uncharacteristic chest pain), dyspnea (e.g., wheezing, coughing, shortness of breath), syncope, palpitations, and seizures. Unspecific symptoms in the form of general malaise and fatigue were also recorded. Symptoms were divided into antecedent and prodromal symptoms. We defined prodromal symptoms as symptoms occurring <24 hours before death and antecedent symptoms as those occurring within a year to 24 hours before death, with the exception of syncope and seizure, which were recorded lifelong, and general malaise and fatigue, which were recorded within the last seven days before death. If the abovementioned symptoms occurred within the abovementioned time limits and these symptoms led to contact with the GP and/or ED, this was recorded as a healthcare contact.

### Statistics

Incidence rates were calculated using the mean background population as the denominator [17]. All calculations and data analysis were performed using SAS version 9.4 (SAS Institute Inc.). Differences in proportions were compared using the chi-square test or Fisher exact test where appropriate. A two sided p-value ≤0.05 was considered statistically significant.

## Results

### Study population

From 2000–2006, there were 6,629 deaths in Denmark in the age group 1–35 years, of which 625 (10%) were SDs. Of those, we identified 49 persons (8%) who had previously suffered from uncontrolled asthma (Figure 1). Of the 49 persons suffering from uncontrolled asthma, 13 (27%) died following an acute asthma attack, of which 8 were autopsied. The remaining 36 persons died of reasons other than acute asthma attack; thirty-one (63%) persons died a SCD, five were explained SCD, and nine were sudden unexplained deaths. Explained SCD was caused by acute myocardial infarction (n = 2), ruptured aortic aneurism (n = 1), cardiac hypertrophy (n = 1), and myocarditis (n = 1). A total of 17 of the 31 SCD cases were non-autopsied SDs presumed to be SCD.

Five patients died of reasons other than asthma or cardiac failure; three because of pulmonary embolism, one because of an abscess in the frontal lobe, and one due to a subarachnoid hemorrhage.

The 49 SDs in patients with uncontrolled asthma equals an incidence rate of 0.32 per 100,000 person-years, whereas the 13 SDs due to asthma equals an incidence rate of 0.08 per 100,000 person-years in the general population.

### Clinical characteristics and circumstances surrounding death

Table 1 shows the clinical characteristics and the circumstances surrounding death in patients with uncontrolled asthma. The majority were Caucasians (n = 45, 92%), and there was a male predominance (n = 33, 67%). The median age at the time of death was 28 years (range 1–35 years), and the mean body mass index (BMI) was 28.9 kg/m^2^.

**Table 1:** **Clinical characteristics and circumstances surrounding death**

**Clinical characteristics (n = 49)**	**n (%)**
Caucasians/Danish ethnicity	45 (92)
Median age at the time of death, years (range)	28 (1–35)
Female gender	16 (33)
Mean BMI* (range)	28.9 (19–55)
Witnessed death	23 (47)
Activity before death	
Respiratory distress	21 (43)
Awake and relaxed	15 (31)
Sleeping	9 (18)
Physical activity	2 (4)
Not specified	2 (4)
Place of death	
Home	25 (51)
Public place	17 (35)
Hospital/ambulance	7 (14)

*Body mass index (kg/m^2^) at the time of autopsy.

**Figure 2:**
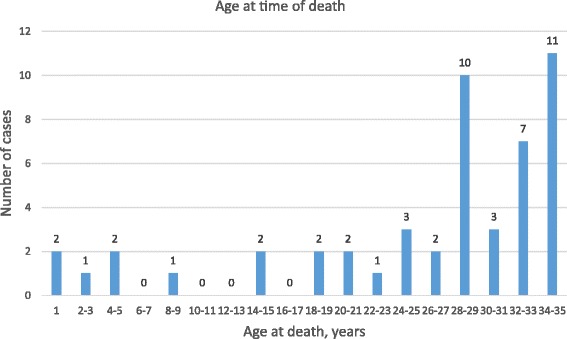
Age distribution at time of death.

There was a trend toward increasing frequency of SD with uncontrolled asthma with increasing age (Figure 2). The place of death was predominantly at home (n = 25, 51%), and the deaths were witnessed in 23 cases (47%). The activity at the time of death was mainly suffering from respiratory distress (n = 21, 43%) followed by awake and relaxed (n = 15, 31%).

Nearly half (n = 24, 49%) of the patients had a previously known disease (in addition to asthma), with psychiatric disorders being the most common (n = 10, 20%) (Table 2). Fourteen men and 10 women had a previously known disease (63% vs. 42%, respectively, p = 0.23).

**Table 2:** **Medical history**

**Medical history (n = 49)**	**n (%)**
Previously known medical history	
No known disease other than asthma	25 (51)
Psychiatric disorder	10 (20)
Epilepsy	4 (8)
Diabetes mellitus	4 (8)
Cardiac disease	4 (8)
Other	3 (6)
Gastritis or reflux	2 (4)
Previously known medical history according to sex	
Men (n = 33)	14 (42)
Women (n = 16)	10 (63)
Abuse	
Drug abuse	10 (20)

Drug abuse was described in 10 (20%) of the cases. It should be emphasized that none of the patients had a toxicology profile that the forensic pathologist concluded could explain their death.

### Symptoms prior to death

Overall, 41 (84%) of the patients had symptoms prior to death; 34 (69%) had antecedent symptoms, whereas 28 (57%) had prodromal symptoms. The most common antecedent symptoms were dyspnea (n = 25, 51%) followed by chest pain (n = 8, 16%). The most common prodromal symptoms were dyspnea (n = 22, 45%) followed by general malaise and/or fatigue (n = 7, 14%) (Table 3).

**Table 3:** **Symptoms prior death**

**Symptoms prior death (n = 49)**	**n (%)**
Overall symptoms	41 (84)
Antecedent symptoms	
Overall symptoms	34 (69)
Dyspnea	25 (51)
Chest pain	8 (16)
Seizures	7 (12)
General malaise and/or fatigue	4 (8)
Syncope	3 (6)
Palpitations	1 (2)
Prodromal symptoms	
Overall symptoms	28 (57)
Dyspnea	22 (45)
General malaise and/or fatigue	7 (14)
Chest pain	1 (2)
Syncope	0 (0)
Palpitations	0 (0)
Seizures	0 (0)
Overall symptoms (excluding solely suffering from dyspnea)	18 (37)

Excluding those patients solely suffering from dyspnea, 18 (37%) had symptoms prior to death.

### Contact with the healthcare system

More than half of the patients (n = 28, 57%) had contact with either their GP and/or the ED prior to SD (Table 4). Seventeen of the patients had contact with their GP, and 16 patients had contact with the ED, of which five had had contact with their GP initially. The most common symptom leading to contact with the GP and/or ED was dyspnea, as expected; however, the type and severity of dyspnea could not be further specified based on the medical records.

**Table 4:** **Healthcare contact**

**Symptom leading to contact with GP and/or ED (n = 28)**	**n**	**Admitted to hospital for further examination (n)**	**Mean number of days before death**
Symptoms leading to contact with the GP	17		
Dyspnea	14	3	191
Uncharacteristic chest pain	3	0	83
Chest tightness	2	1	3
General malaise and fatigue	2	0	0-1
Syncope	1	1	4676
Palpitations	0	0	-
Symptoms leading to contact with the ED	16		
Dyspnea	11	-	85
Syncope	3	-	2195
Uncharacteristic chest pain	1	-	161
Chest tightness	1	-	3
Palpitations	0	-	-
General malaise and fatigue	0	-	-

### Asthma medication use

In total, 49% of patients (n = 24) used asthma medication at the time of death, with the combination of inhaled corticosteroids and short-acting beta-adrenoceptor agonists the most common (n = 9, 18%) (Table 5). None of the patients were taking long-acting beta-adrenoceptor agonists only. No registered use of asthma medication was reported in eight patients (16%). We had insufficient data on the medical intake of asthma medicine for nearly one-third of patients (n = 17, 35%).

**Table 5:** **Asthma medication**

**Asthma medication information (n = 49)**	**n (%)**
No asthma medication	7 (14)
Asthma medication	
SABA	7 (14)
LABA + SABA + ICS	3 (6)
ICS	1 (2)
ICS + SABA	9 (18)
ICS + LABA	2 (4)
SABA + LABA + ICS + ML	2 (4)
Unknown	18 (37)

SABA = short-acting beta-adrenoceptor agonist.

LABA = long-acting beta-adrenoceptor agonist.

ICS = inhaled corticosteroid.

ML = montelukast.

### Sub-analysis of patients ≤ 5 years old

A sub-analysis on the clinical characteristics and symptoms of the children ≤ 5 years is available as Additional file 1: Table S1 and S2). In our study, the children ≤ 5 years all suffered from antecedent symptoms (with dyspnea being predominant), and four (80%) suffered from prodromal symptoms (of which dyspnea was predominant).

## Discussion

### Incidence of asthma

This is the first nationwide study to characterize clinical characteristics and symptoms prior to SD in patients aged 1–35 years (n = 49) with uncontrolled asthma.

Following a systematic review of all death certificates, information from the National Patient Registry, and all available medical records from hospitals and GPs, we report that acute asthma attacks were the cause of death in 13 of 625 (2.1%) SD cases. Forty-one (84%) had symptoms prior to death, of which dyspnea was predominant, and 28 (57%) sought medical advice for these symptoms.

The incidence of fatal asthma attacks reported in the literature has varied significantly. Data based on death certificates from out-of-hospital deaths in persons younger than 40 years recorded in the Netherlands found asthma to be the cause of death in 37 out of 1908 SDs (1.9%) [18]. This result is consistent with the data of our study. However, another retrospective study [19] that investigated 151 cases of SD among Israeli military personnel at the age of 18 to 39 years found that fatal asthmatic attacks were the cause of death in 9% of all SD cases. The soldiers being exposed to occupational and environmental hazards that increased the risk of fatal asthma attacks could in part explain the high incidence of fatal asthma attacks in their study.

### Clinical characteristics

We report a high mean BMI value (28.9 kg/m^2^). However, this is consistent with a recent study from our group on SCD due to coronary artery disease (age 1–35 years), in which we also report a high mean BMI of 26.4 kg/m^2^ [14].

The presence of excess abdominal fat decreases lung volume and affects the function of the diaphragm (increasing the likelihood of it suffering fatigue during stressful activity), and it has also been demonstrated that obesity increases the risk of suffering from uncontrolled asthma [20]. It is therefore likely that the high mean BMI in our study increased the severity of the patients’ asthma and may have influenced their death.

### SCD and asthma

SCD in persons aged 1–35 corresponds to approximately 75% of all SDs in this age group [8].

Our results indicate that 63% of the SDs of patients with uncontrolled asthma are due to SCD, which is actually lower than in the general population. This may be attributed to the fact that a high proportion of fatal asthma attacks was reported as the cause of death in our study.

### Medical history

We report that a seemingly large proportion of the patients (n = 24, 49%) had a previously known disease besides asthma. It has been reported that SD at an age of 1–20 years was attributed to a previously diagnosed condition in 53% of the SD cases [21]. When asthma diagnoses were excluded from this previous study, 41% had a previously known disease, which is similar to the results of our study.

Poor adherence to medication in patients with asthma has been shown to be significantly increased when psychological morbidity is present [22]. According to our study, 20% of the patients had been diagnosed with a psychiatric disease, which appears to be rather high. However, recent data based on interviews with 21,425 persons > 18 years old from six different European countries showed (consistent with our findings) that one in four of the respondents reported a lifetime occurrence of any mental disorder [23]. Furthermore, we have also reported that psychiatric disorders were prevalent in 18% of a randomly selected group of young persons who died in accidents [14].

### Symptoms prior to death

We report a high prevalence of symptoms prior to death (n = 41, 84%), of which dyspnea was predominant. In contrast to our findings, it has previously been reported that 35% of SDs among military personnel aged 18 to 39 years in the Israeli army had symptoms at some point prior to death [19]. These symptoms included syncope, chest pain, palpitations, recent febrile disease, dyspnea, gastrointestinal symptoms, headache, and visual disturbances (in the prior two weeks). Another study [24] reported prodromal symptoms in 54% of persons < 40 years old suffering from SD. Prodromal symptoms in this study had no time limit before death, in contrast to our study, and symptoms were defined as any change from normal health status that was considered noteworthy by the subject and reported to family, friends or medical authorities. However, it is notable that subjects with previously diagnosed underlying diseases (such as asthma) were excluded from the study.

The high prevalence of symptoms prior to death in our study compared with the abovementioned studies can be attributed to the high occurrence of dyspnea in our study, which is naturally expected to be present in a population consisting of young persons with uncontrolled asthma.

If we exclude the persons that exclusively suffered from dyspnea in our study, 18 (37%) had symptoms prior to death, which is more consistent with the abovementioned studies.

Although we extracted information from all possible sources, the amount of prodromal symptoms might have been underestimated due to only 53% of the SDs being unwitnessed.

### Contact with the healthcare system

We found that 41 of patients (84%) had symptoms prior to death and that just half of the patients sought medical attention, of which 17 (35%) sought medical advice from their GP and 16 (33%) from the ED. In a study by Tough et al. [25], it was previously reported that 87% of patients had contact with the hospital prior to death, which appears rather high compared with our study. There is, however, a discrepancy in the design of the study by Tough et al. compared with ours. Tough et al. analyzed death in patients who had died solely of asthma (not necessarily defined as SD), whereas our study focuses on individuals who have been categorized as suffering from uncontrolled asthma prior to death. Furthermore, the hospital records in our study focused on the 12 months prior to death, whereas the study by Tough et al. included all previous hospital contact.

### Asthma medication

It is surprising that seven patients (14%) were not taking any asthma medicine at the time of death. This may be attributed to low compliance. A recent meta-analysis concluded that monotherapy of long-acting beta-adrenoceptor agonists is associated with an increased risk of asthma mortality compared with the combination of long-acting beta-adrenoceptor agonists and inhaled corticosteroids [26]. However, none of the patients in our study used long-acting beta-adrenoceptor agonists as a monotherapy.

### Study strengths and limitations

It is a limitation of our study that data were collected retrospectively. The results of our study can only be applied to persons having suffered from uncontrolled asthma prior to the event and therefore not asthma in general. Furthermore, on the basis of the available medical records, we were not able to better classify the symptomology with regards to dyspnea, chest pain, general malaise and fatigue. If more precise medical records were available, it would have been interesting to subdivide the different symptoms into, for example, mild, moderate, and severe, to better assess the symptoms.

The current study design does not allow for the identification of independent risk factors associated with uncontrolled asthma.

Asthma is very difficult to diagnose, particularly in the first early years of life because asthma masquerades are common.

A sub-analysis of the infants (≤5 years) is available as supplementary data. However, because of the low number of patients (n = 5) in this age group, a comparison between them and patients > 5 years would be uninformative.

Instead of solely relying on data from autopsy reports and death certificates, we furthermore retrieved and reviewed all available patient records from the GPs, and using the civil registration number, we retrieved information on prior medical history from the National Patient Registry. Information from the GP was obtained for 33 patients (67%), and together with the information from the National Patient Registry and the Danish death certificates, we therefore have very reliable combined resources to describe the 49 SD cases diagnosed with uncontrolled asthma.

## Conclusion

SD in the young is a rare event. In this retrospective study, 49 persons with uncontrolled asthma suffered an SD, corresponding to 8% of all SDs. The majority (63%) died a SCD; 27% died due to an asthma attack.

In total, 84% of the persons with uncontrolled asthma had symptoms before death, of which dyspnea was the most predominant symptom. A large proportion had other diseases in addition to asthma (49%), of which psychiatric disorders were predominant. On the basis of symptoms prior to death, 17 (35%) and 16 (33%) of the patients sought medical advice from their GP and/or an ED, respectively.

It is difficult to identify young persons with uncontrolled asthma with a high risk of SD. However, by studying the symptoms before SD, it may be possible to identify risk factors associated with this devastating event. We hope that this will increase the medical focus on young persons with uncontrolled asthma and thereby aid in preventing SDs.

## Additional file

Additional file 1:
**Supplementary data on infants** ≤ **5 years.** **Table S1.** – Clinical characteristics and circumstances surrounding the death for infants ≤ 5 years of age. **Table S2.** Symptoms prior to death in infants ≤ 5 years of age.

## Abbreviations

SD: Sudden unexpected death
SCD: Sudden cardiac death
GP: General practitioner
ED: Emergency department
BMI: Body mass index
