# Supplementary material for: Sudden death in young persons with uncontrolled asthma - a nationwide cohort study in Denmark
Source: BMC Pulm Med. 2015 Apr 14;15:35. doi: 10.1186/s12890-015-0033-z (PMC4404085; doi:10.1186/s12890-015-0033-z)
Supplement: Additional file 1: — Supplementary data on infants ≤ 5 years. Table S1. – Clinical characteristics and circumstances surrounding the death for infants ≤ 5 years of age. Table S2. Symptoms prior to death in infants ≤ 5 years of age. [file 12890_2015_33_MOESM1_ESM.docx]

**SUPPLEMENTAL MATERIAL**

Sudden death in young persons with uncontrolled asthma

- a nationwide cohort study in Denmark

**Anders Juul Gullach, MD^1,2*^; Bjarke Risgaard, MD^1,2^; Thomas Hadberg Lynge, BM^1,2^; Reza Jabbari, MD^1,2^; Charlotte Glinge, BM^1,2^; Stig Haunsø, MD, DMSc^1,2,3^; Vibeke Backer MD DMSc^4^; Bo Gregers Winkel, MD, PhD^1,2^ and Jacob Tfelt-Hansen, MD,DMSc^,2,3^**

**Affiliations** ^1^Danish National Research Foundation Centre for Cardiac Arrhythmia (DARC), University of Copenhagen, Copenhagen, Denmark**;** ^2^Laboratory of Molecular Cardiology, Department of Cardiology, The Heart Centre, Copenhagen University Hospital, Rigshospitalet, Copenhagen, Denmark**;** ^3^Department of Medicine and Surgery, University of Copenhagen, Copenhagen, Denmark; ^4^Department of Respiratory Medicine, Bispebjerg University Hospital, Copenhagen, Denmark

**Page 2:** eTable 1: Clinical characteristics and circumstances surrounding the death for infants ≤ 5 years of age

**Page 3:** eTable 2: Symptoms prior to death in infants ≤ 5 years of age

**Supplementary data on infants ≤ 5 years**

*eTable 1 – Clinical characteristics* *and circumstances surrounding the death for infants ≤ 5 years of age*

| **Clinical characteristics (n=5)** | **n (%)** |
| --- | --- |
| Caucasians/Danish ethnicity | 5 (100) |
| Median age at the time of death, years (range) | 2,8 (1-5) |
| Female gender | 2 (40) |
| Mean BMI*, (range) | 19,3 |
| Witnessed death, | 2 (40) |
| Activity before death, |  |
| Respiratory distress | 2 (40) |
| Sleeping | 2 (40) |
| Not specified | 1 (20) |
| Place of death, |  |
| Home | 3 (60) |
| Hospital/ambulance | 2 (40) |

* Body Mass Index (kg/m^2^) at the time of autopsy

*eTable 2. Symptoms prior to death in infants ≤ 5 years of age*

| **Symptoms prior death (n=5)** | **n (%)** |
| --- | --- |
| Overall symptoms | 5 (100) |
|  |  |
| Antecedent symptoms |  |
| Overall symptoms | 5 (100) |
| Dyspnea | 3 (60) |
| Chest pain | 0 (0) |
| Seizures | 2 (40) |
| General malaise and/or fatigue | 0 (0) |
| Syncope | 0 (0) |
| Palpitations | 0 (0) |
|  |  |
| Prodromal symptoms |  |
| Overall symptoms | 4 (80) |
| Dyspnea | 3 (60) |
| General malaise and/or fatigue | 1 (20) |
| Chest pain | 0 (0) |
| Syncope | 0 (0) |
| Palpitations | 0 (0) |
| Seizures | 0 (0) |
|  |  |
| Overall symptoms (excluding solely suffering from dyspnea) | 3 (60) |
